# Use of Electronic Nicotine Delivery Systems (ENDS) by pregnant women I: Risk of small-for-gestational-age birth

**DOI:** 10.18332/tid/106089

**Published:** 2019-05-21

**Authors:** Victor M. Cardenas, Ruiqi Cen, Melissa M. Clemens, Heather L. Moody, Uwemedimbuk S. Ekanem, Anuradha Policherla, Lori A. Fischbach, Hari Eswaran, Everett F. Magann, Robert R. Delongchamp, Gunnar Boysen

**Affiliations:** 1 Department of Epidemiology, Fay W. Boozman College of Public Health, University of Arkansas for Medical Sciences, Little Rock, United States; 2 The Winthrop P. Rockefeller Cancer Institute, University of Arkansas for Medical Sciences, Little Rock, United States; 3 Department of Environmental and Occupational Health, Fay W. Boozman College of Public Health, University of Arkansas for Medical Sciences, Little Rock, United States; 4 Department of Obstetrics and Gynecology, College of Medicine, University of Arkansas for Medical Sciences, Little Rock, United States; 5 Department of Community Health, Faculty of Clinical Sciences, University of Uyo, Uyo, Nigeria; 6 Arkansas Department of Health, Little Rock, United States

**Keywords:** pregnancy, nicotine, cohort studies, electronic nicotine delivery systems, USA

## Abstract

**INTRODUCTION:**

The 2016 US Surgeon General’s Report suggests that the use of electronic nicotine delivery systems (ENDS) is a fetal risk factor. However, no previous study has estimated their effect on adverse pregnancy outcomes. We assessed the prevalence of current ENDS use in pregnant women and explored the effect on birth weight and smallness-for-gestational-age (SGA), correcting for misclassification from nondisclosure of smoking status.

**METHODS:**

We conducted a cohort study with 248 pregnant women using questionnaire data and biomarkers (salivary cotinine, exhaled carbon monoxide, and hair nicotine). We evaluated the association between birth weight and the risk of SGA by applying multivariate linear and log-binomial regression to reproductive outcome data for 232 participants. Participants who did not disclose their smoking status were excluded from the referent group. Sensitivity analysis corrected for misclassification of smoking/ENDS use status.

**RESULTS:**

The prevalence of current ENDS use among pregnant women was 6.8% (95% CI: 4.4–10.2%); most of these (75%) were concurrent smokers. Using self-reports, the estimated risk ratio of SGA for ENDS users was nearly two times the risk in the unexposed (RR=1.9, 95% CI: 0.6–5.5), and over three times that for ENDS-only users versus the unexposed (RR=3.1, 95% CI: 0.8–11.7). Excluding from the referent group smokers who did not disclose their smoking status, the risk of SGA for ENDS-only use was 5 times the risk in the unexposed (RR=5.1, 95% CI: 1.1– 22.2), and almost four times for all types of ENDS users (RR=3.8, 95% CI: 1.3–11.2). SGA risk ratios for ENDS users, corrected for misclassification due to self-report, were 6.5–8.5 times that of the unexposed.

**CONCLUSIONS:**

Our data suggest that ENDS use is associated with an increased risk of SGA.

## INTRODUCTION

A 2013–2014 US national study found that 4.9% of 388 pregnant women were current users of electronic nicotine delivery systems (ENDS); most (79.4%) were also current cigarette smokers (i.e. dual ENDS users)^1^, consistent with findings of national surveys of adults in the US^2-4^. ENDS use has increased steadily since their introduction to the US market in 2007^5^; ENDS include electronic cigarettes, cartomizers, atomizers as well as the novel JUUL device^6,7^. The consequences of ENDS use during pregnancy remain largely unknown, as expressed in a recent review by the US NationalAcademy of Sciences^8^. A systematic review on the topic, we conducted in October 2017 and updated in November 2018, found no human studies as knowledge base for practice recommendations and the education of professionals^9^. However, the 2016 US Surgeon General’s Report on ‘E-Cigarette Use Among Youth and Young Adults’ reported that ‘the effects of nicotine and the potential for harm by other e-cigarette toxicants indicate that the use of ENDS is a fetal risk factor’^10^.

It is not clear whether nicotine alone from ENDS is associated with fetal growth restriction, as inferred from studies of maternal smokeless tobacco use^11,12^. The tobacco industry markets ENDS as ‘healthier’ than cigarette smoking^13^ because there is considerably less hazardous material in ENDS aerosols than in cigarette smoke, and ENDS do not generate products of combustion such as carbon monoxide. Some pregnant women and some healthcare providers believe that ENDS use is less harmful than cigarette smoking and may even reduce cigarette smoking during pregnancy^14-21^. However, most obstetricians, advice against the use of ENDS during pregnancy: in 2017, the American College of Obstetricians and Gynecologists recognized the paucity of data on the health effects of ENDS use during pregnancy but found no evidence to support ENDS use as a smoking cessation aid^22^.

With few exceptions, previous epidemiologic studies of smoking, fetal weight, and small-for-gestational-age (SGA) have appraised the relation between smoking and SGA using self-reports for smoking^23^. However, pregnant women are 2.5 times more likely than non-pregnant women to underreport smoking, according to a US national study that found that 23% of pregnant smokers do not disclose their smoking habit^24^. Correction of the association between smoking and the risk of SGA for the misclassification introduced by the error of self-report of smoking status using urinary cotinine, decreased the risk estimate of SGA for smokers in one study^25^, but the life-time of urinary cotinine is only 16 hours^26^. Nicotine in hair is a validated biomarker for past active or passive smoking; hair nicotine has a longer half-life than other biomarkers such as plasma, urinary or salivary cotinine, and just 3 cm of hair from close above the scalp is required for the assay to assess the exposure in the past three months^27^. Previous studies that measured hair nicotine among pregnant participants, had reported larger reductions in z-scores of birth weight and a larger increased risk of SGA^28-30^. However, no studies have included pregnant women who used ENDS.

The Behavioral Risk Factor Surveillance System (BRFSS) estimated the prevalence of smoking in adults in Arkansas to be 24.8% in 2014, among the highest in the nation^31,32^. Given the high-risk for ENDS use, we had analyzed data from the Arkansas BRFSS on ENDS use and found that 6.1% of adults reported using ENDS within the past month^33^. Considering highly relevant a study to describe the prevalence of current ENDS use in pregnant women, we aim to: 1) assess the validity of self-reported ENDS use and cigarette smoking using salivary cotinine, exhaled carbon monoxide (CO), and hair nicotine as gold-standards; 2) examine the association of ENDS use during pregnancy with birth weight and the risk of SGA; and 3) reassess the association with the risk of SGA correcting for misclassification by self-report of tobacco use.

## METHODS

### Design

For this pregnancy cohort study, we recruited volunteers among patients seen at a prenatal clinic serving low-risk pregnant women (i.e. those without underlying medical conditions or co-morbidities and without antenatal complications) and assessed their exposure to tobacco products by self-report and non-invasive biomarker assays. We also obtained permission to access their medical records to extract specific data on the reproductive outcomes described below.

### Participants

Our study population consisted of pregnant women seeking prenatal care at a low-risk pregnancy clinic of a University affiliated center in Little Rock, Arkansas. The clinic is a low-risk pregnancy clinic, i.e. it provides care to ‘singleton, term, vertex pregnancies, (without) any other medical or surgical conditions’^34^. Pregnant women were eligible if they were ≥18 years old, spoke English, and planned to deliver their babies at the University affiliated hospital. Patients from the teen pregnancy clinics and high-risk patient clinics were therefore not included. From April 2015 to May 2017, eligible pregnant women were queried to identify smokers and ENDS users. From November to December 2016, the recruitment was non-consecutive, instead we identified and enrolled an ENDS user first, followed by the next smoker, and then the next nonsmoker. The questions were previously developed by Mullen et al.^35^ to improve disclosure of smoking status among pregnant women. We added a similar question to identify ENDS users.

### Data collection

Participants were asked to fill in a 10-minute self-administered questionnaire assisted with a tablet computer using an application developed with LimeSurvey (GmbH, Hamburg, Germany). The questionnaire collected data on ever and current cigarette smoking and use of other tobacco products, including ENDS, the time since their last use, and their exposure to secondhand smoke/ENDS aerosol. In 2016, we added a question about the number of cigarettes smoked in the 3 months before the current pregnancy; therefore, this information was limited to a subset of participants. We also asked the participants to provide a 2 mL sample of saliva through a funnel into a vial for on-site testing of salivary cotinine (NicAlert, Nymox, St. Laurent, Quebec). According to the manufacturer, the cutoff value of this test for tobacco use is ≥10 ng/mL. Exhaled CO levels were collected by asking the participants to take a deep breath, hold it for 10 seconds, and breathe out slowly through a cardboard mouthpiece into a babyCOmpact, Smokerlyzer unit (Bedford Scientific, Haddonfield, NJ). According to the manufacturer, the cutoff value of CO to identify smoking is ≥7 ppm.

Because cotinine in fluids such as saliva has a short half-life (16 hours)^26^, and previous studies demonstrated that hair nicotine is a more reliable biomarker of long-term exposure^27^, particularly for reproductive outcomes from maternal exposure to tobacco, we measured hair nicotine levels as described in the companion manuscript^36^.

Ever users of ENDS were defined as those who reported that they had tried ENDS, and current users were defined as those who reported ENDS use within the previous month. Similarly, ever cigarette smokers were defined as those who reported smoking at least 100 cigarettes in their lifetime, current cigarette smokers were defined as those who reported smoking in the previous month. Thus, we classified the participants according to self-report into one of the following six groups: 1) current ENDS dual users including concurrent cigarette smokers, 2) current ENDS-only users, 3) current cigarette smokers who currently did not use ENDS, 4) non-current smokers/non-current ENDS users not exposed to secondhand smoke or ENDS aerosols or other tobacco products, 5) non-users of tobacco products but exposed to secondhand smoke or ENDS aerosols, and 6) users of tobacco products other than cigarettes or ENDS. Among the non-current smokers there were only two ever smokers who reportedly stopped smoking more than a year before. Because the most likely threat to the validity of our study would be a measurement error introduced by misclassification due to nondisclosure of smoking status, we used data from salivary cotinine or CO tests to exclude undisclosed active tobacco users from the referent group (i.e. the fourth group listed above).

We obtained each neonate’s estimated gestational age at birth and birth weight from medical records. We then used the US National Center for Health Statistics birth data as referent^37^, obtaining gender- and gestational age-adjusted z-score for birth weight for each singleton birth in our study population. Furthermore, we used the 10th percentile of the gender-specific and gestational age-specific birth weight^38^ to identify SGA.

### Ethical considerations

The protocol was approved by the Institutional Review Board (Protocol Number 203805) of the authors’ University. Participants who reported using tobacco and wanted to quit were provided with a flyer with a toll-free number to a smoking-cessation resource. We obtained written informed consent from the participants to: collect questionnaire data, breath, saliva, and hair specimens for markers of tobacco use; access the participants’ personal prenatal medical records; and retrieve specific data from their medical and birth records.

### Data analysis

The association of ENDS use with age, income, education, occupation, weeks of gestation (if known at baseline) and cigarette smoking was assessed using the entire set of observations. We compared the self-reported levels of smoking and ENDS use along with the distribution of hair nicotine, salivary cotinine, and CO, in each of the six comparison groups. We used the z-score of the birth weight of the participants’ neonates as a continuous outcome variable, while SGA was treated as a dichotomous outcome variable. Confidence intervals (CIs) around proportions were calculated using the Wilson score method^39^. Stratified analyses were used to adjust the risk ratio (RR) using the Mantel-Haenszel estimator of the common RR^40^. Multiple regression analyses were conducted for birth-weight data, while multiple logistic regression analysis was performed for SGA using the log-binomial model to estimate the RR and its 95% CI^41^, as the outcome (SGA) was common (>10%) in the study population. We conducted sensitivity analyses to correct the estimate of the size of the association between tobacco use and the risk of SGA for misclassification by self-report of smoking/ENDS use. Specifically, we used two approaches for this. First, we excluded from the referent group those self-reported non-users of tobacco not exposed to secondhand smoke or ENDS aerosols who had salivary cotinine or CO levels consistent with active smoking/ENDS use. Second, we used the estimates of sensitivity and specificity for self-report of smoking using hair nicotine as the gold-standard, both from our own study population and from estimates published in the literature^29^, which were then applied for correction of misclassification of self-report, using the formula described elsewhere^42^. We also considered other pregnancy outcomes such as preterm delivery (PTD, i.e. a neonate delivered at less than 37 weeks of gestation) and admissions to the neonatal intensive care unit. However, in this low-risk pregnancy clinic study population, there were few PTDs and other adverse reproductive outcomes (Supplementary Table 1), and we focused our assessment on the adjusted z-score for birth weight and SGA. The sample size estimates were based only on the estimation of the prevalence of ENDS use and were deemed exploratory for the remaining study objectives. All of these analyses used complete case analysis and were conducted with SAS v9.4 (SAS Institute, Cary, NC).

**Table 1:** Frequency of current use* of electronic nicotine delivery systems (ENDS) among pregnant women by age, weeks of gestational age at enrollment, parity, race/ethnicity, education, income, and current cigarette smoking in Little Rock, Arkansas, 2015–2017 (N=248)

*Characteristics*	*Current ENDS Use*		*Current ENDS*	p**
	*Yes*	*Total N (Column %)*	*Row Per cent*	
***Age*** *(years)*				
18–22	6	94 (37.9)	6.4	0.20
23–27	11	76 (30.6)	14.5	
≥28	7	78 (31.5)	9.0	
Total	24	248 (100.0)	9.7	
**Weeks of gestation at enrollment**				
< 20	12	84 (33.9)	14.3	0.03
≥20	11	162 (65.3)	6.8	
Missing	1	2 (0.8)	50.0	
Total	24	248 (100.0)	9.7	
**Parity**				
0	9	99 (39.9)	9.1	0.9
1	7	61 (24.6)	11.5	
≥2	8	88 (35.5)	9.1	
Total	24	248 (100.0)	9.7	
**Race/Ethnicity**				
Non-Hispanic Blacks	7	112 (45.2)	6.3	0.01
Non-Hispanic White	15	95 (38.3)	15.8	
Hispanic	0	30 (12.1)	0.0	
Other	2	11 (4.4)	18.2	
Total	24	248 (100.0)	9.7	
**Education**				
Below High School level	7	60 (24.2)	11.7	0.61
High School level and above	17	188 (75.8)	9.0	
Total	24	248 (100.0)	9.7	
**Annual household income** (US$)				
≤15000	12	91 (36.7)	13.2	0.39
>15000	5	67 (27.0)	7.5	
Missing	7	90 (36.3)	7.8	
Total	24	248 (100.0)	9.7	
**Employment**				
Employed	7	101 (40.7)	6.9	0.38
Student	1	21 (8.5)	4.8	
			
Homemaker	4	24 (9.7)	16.7	
Not working	12	102 (41.1)	11.8	
Total	24	248 (100.0)	9.7	
Current cigarette smoking*				
Yes	18	77 (31.0)	23.4	<0.0001
No	6	171 (69.0)	3.5	
Total	24	248 (100.0)	9.7	

* Reported use within the last month.

** Fisher’s exact two-tailed p-value.

## RESULTS

### Recruitment and follow-up

**Figure 1:**
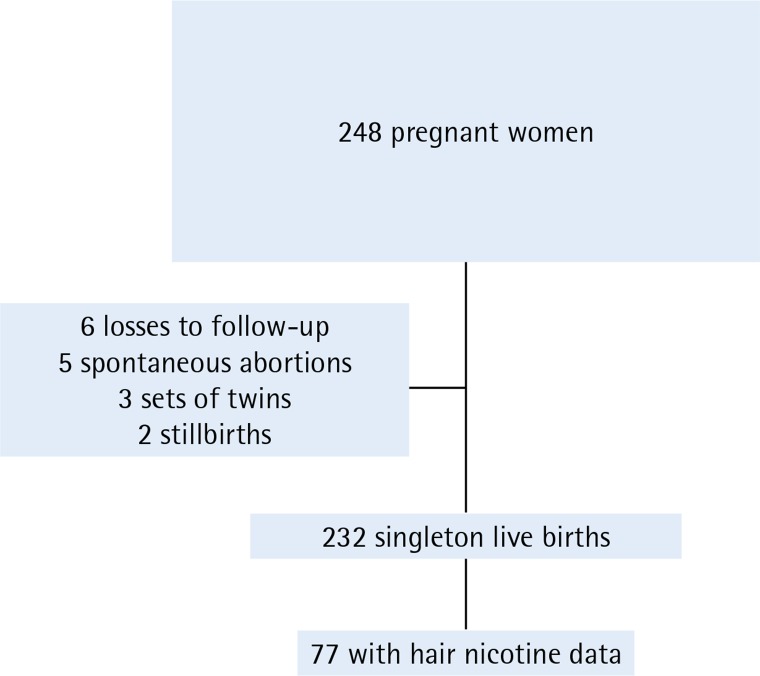
Flow chart of pregnancy cohort study of electronic nicotine delivery systems use and pregnancy outcomes, Little Rock, Arkansas, 2015-2017

From April 2015 to May 2017, we enrolled 248 pregnant women in our study, and only 41 were enrolled non-consecutively. By 29 November 2017, we had followed 242 pregnancies (97.6%) to the end of gestation. The participants were 23.0% of the approximately 1080 pregnant women seeking prenatal care at the low-risk pregnancy clinic during the study period. After excluding 5 spontaneous abortions, 2 stillbirths, 3 sets of twins, and 6 pregnancies with missing outcome data, there were 232 singleton live births included in the analysis of reproductive outcomes. We obtained saliva and breath samples for all participants. We were able to test hair specimens in a subset of 81 participants and among these 77 had complete data through the end of gestation (Figure 1).

The characteristics of the study population are shown in Table 1. Almost 38% of the participants were less than 23 years old, and 65.3% were enrolled during the second half of their pregnancy. Moreover, 45.2% were non-Hispanic Blacks, 38.3% were non-Hispanic Whites, 12.1% were Hispanic, and 4.4% belonged to other racial/ethnic groups. Twenty-four per cent did not graduate from high school, thirty-seven per cent lived in households with an annual income of ≤ $15000, 4.11% reported not working, and 31.0% reported smoking cigarettes.

### Prevalence of ENDS use

Of the 248 enrolled participants, 207 were selected using consecutive sampling; 14 were current ENDS users, corresponding to an estimate of 6.8% (Wilson 95% CI: 4.4–10.2%) of current ENDS use. The prevalence of exclusive ENDS use was estimated to be 2.4% in the consecutive sample (5/207) (Wilson 95% CI: 1.1–4.7%), but only 1 (0.5%) participant reported using ENDS daily (Wilson 95% CI: 0.1–2.1%). The prevalence of current cigarette smoking in the consecutive sample was 27.0% (or 56/207) (Wilson 95% CI: 22.3–32.7%).

Overall, among the 248 participants, there were 24 (9.7%) current ENDS users; 18 (75%) were dual users; 59 (23.8%) were current cigarette smokers who did not use ENDS; 47 (19.0%) who did not use any tobacco product but were exposed to smoke or ENDS aerosols; 106 (42.7%) who did not smoke or use tobacco nor exposed to secondhand smoke or ENDS aerosols of others; and 12 (4.8%) who reported chewing tobacco or smoking cigars or pipes, but not using cigarettes or ENDS.

### Frequency of ENDS use

The frequency of self-reported ENDS use among the 24 current ENDS users was: daily to 10 days a month for 5 users (20.8%), 3 to 9 times a month for 7 users (29.2%), and 1 to 2 times a month for the remaining 12 users (50%).

### Risk factors for current ENDS use

We did not observe an association between current ENDS use and the following covariates: age, education, annual income, occupation, employment or parity (Table 1). However, we did observe associations between ENDS use and race/ethnicity, weeks of gestation at enrollment, and a particularly strong association with current cigarette smoking. The prevalence of current ENDS use among Non-Hispanic Whites (15.8%) was more than twice the prevalence among non-Hispanic Blacks (6.3%). The prevalence of current ENDS use in participants who reported as ‘other’ as their race/ethnicity was estimated to be 18.2%, almost 3 times that of non-Hispanic Blacks. Finally, none of the 30 Hispanic participants reported current ENDS use. Current cigarette smoking was reported by 18/24 current ENDS users (75.0%); while, 23.4% of current cigarette smokers also currently used ENDS, compared with only 3.5% of non-current cigarette smokers (p<0.0001). The most important risk factor for ENDS use was cigarette smoking.

### Smoking history, cigarette consumption and current ENDS use

We looked into the past history of smoking because a large proportion of pregnant women spontaneously stop smoking: half of the ENDS-only users (n=3) and one-fifth (21.2% or n=35) of non-current cigarette smokers were ever cigarette smokers. Most (15/18, 83.3%) of the dual ENDS users reported that they tried to stop smoking cigarettes, while 66.1% (or 39/59) of current cigarette smokers (at baseline) who did not use ENDS reported that they tried to stop smoking cigarettes; however, the difference was not statistically significant (p=0.174).

Data on the number of cigarettes smoked in the 3 months before pregnancy were available for 173 of the participants. In this subset, 76 reported smoking at least 1 cigarette per day before they knew they were pregnant. Fifty-four (71.0%) reported smoking ≥6 cigarettes per day in the 3 months before their pregnancy, but only 30 (39.5%) reported smoking ≥6 cigarettes per day at enrollment, hence 24 (44.4%) decreased below such level. There were no instances of increased number of cigarettes smoked, and all 22 participants who reported smoking ≤5 cigarettes per day in the 3 months before pregnancy continued smoking ≤5 cigarettes per day at enrollment (RR=0.444, 95% CI: 0.3–0.6; Fisher’s exact test p<0.0001). The decrease of self-reported number of cigarettes smoked did not change according to use of ENDS.

At baseline, ENDS dual users smoked an average of 6.3 cigarettes per day (SD=4.7), while non-ENDS users smoked an average of 7.3 cigarettes per day (SD=5.6) (t-test p=0.4). ENDS users who also smoked cigarettes (i.e. dual ENDS users) decreased their smoking during pregnancy by 9.6 cigarettes per day, compared with the number smoked 3 months before pregnancy (-9.6, standard error SE=2.2). Similarly, smokers who did not use ENDS decreased their smoking during pregnancy by almost 8 cigarettes per day at baseline, compared to the number smoked 3 months before pregnancy (-7.8, SE=1.4). The difference in the reduction of cigarettes smoked between ENDS dual users and smokers who were non-current ENDS users was not statistically significant (Satterthwaite t-test p=0.5).

### Salivary cotinine, CO, hair nicotine, and cigarette smoking/ENDS use

Data on salivary cotinine were available for all 248 participants. The proportion of ENDS dual users, ENDS-only users, and smokers with salivary cotinine values above the manufacturer’s recommended cutoff value of ≥10 ng/mL to distinguish active smokers, were 83.3%, 83.3%, and 98.3%, respectively. The sensitivity and specificity of the self-report of smoking or ENDS use for a cutoff of ≥10 ng/mL salivary cotinine were 58.2% (95% CI: 49.7–66.2%) and 90.9% (95% CI: 80.4–96.1%), respectively.

Notably, we also found that 52.8% (56/106) of participants who reported no exposure to tobacco (including secondhand smoke) had salivary cotinine levels above the cutoff value of ≥10 ng/mL. Those 56 self-reported unexposed participants with salivary cotinine levels ≥10 ng/mL represent 38.3% of all active tobacco users (n=146) who did not disclose their active tobacco-use status.

The proportion of ENDS dual users, ENDS-only users, and smokers with CO above the cutoff value of ≥7 ppm were 72.2%, 16.7%, and 76.3%, respectively. The corresponding sensitivity and specificity for the self-reported dual ENDS use/smoking (i.e. all exposed to combustible tobacco) were 81.7% (95% CI: 71.2–89.0%) and 83.0% (95% CI: 75.0–88.9%), respectively. Using the CO cutoff of 7 ppm, we found that 12.3% (13/106) of self-reported non-smokers did not disclose being smokers; eight (61.5%) of these also had salivary cotinine levels ≥10 ng/mL.

Using a cutoff of ≥2.77 ng/mg of hair nicotine, 81.8% of ENDS dual users and 88.9% of smokers had levels of hair nicotine consistent with active smoking. A large proportion of those who self-reported no exposure to tobacco had hair nicotine levels that were consistent with smoking within the last 3 months (7/29 or 24.1%). The corresponding estimates of the sensitivity and specificity for self-reported active tobacco use, against hair nicotine as standard, were 82.5% (95% CI: 68.1–91.3%) and 84.6% (95% CI: 63.3–91.8%), respectively. Using either short-term biomarkers of tobacco use (salivary cotinine), or short-term combustible tobacco (CO), or a long-term biomarker of tobacco use (hair nicotine), we found extensive nondisclosure of tobacco use ranging from 12.3% for CO to 52.8% for salivary cotinine, with an intermediate value of 24.1% with the marker of long-term use, hair nicotine.

### Birth weight outcomes and ENDS use/smoking status

The pregnancy outcomes for the 232 participants with complete data are presented in Table 2. Only maternal age and race/ethnicity changed the estimates of the effect of ENDS use on birth weight and SGA outcomes by more than 10%. After controlling for maternal age and race/ethnicity, the gender and gestational age-specific birth weight z-scores for ENDS dual users and ENDS-only users were lower than those for non-smokers. Neonates of women who smoked while pregnant had significantly lower gestational age-specific and sex-specific birth weight z-scores than those of self-reported unexposed (-0.482, SE=0.177; p<0.05).

**Table 2:** Pregnancy outcomes of pregnant women according to ENDS and/or smoking status in Little Rock, Arkansas, 2015–2017 (N=232)

*Self-reported tobacco use at baseline*	*n*	Multivariate* mean z-score birth weight difference (SE)	*Smallness-forgestational-age (%)*	*Smallness-forgestational-age crude risk ratio ( 95% CI)*	Smallness-forgestational-age multivariate* risk ratio ( 95% CI)
Current ENDS dual users	17	-0.297 (0.266)	4 (23.5)	2.1 (0.7–5.8)	1.9 (0.6–5.5)
Current ENDS-only users	6	-0.498 (0.411)	2 (33.3)	2.9 (0.8–10.4)	3.1 (0.8–11.7)
Any current ENDS use	23	-0.353 (0.233)	6 (26.1)	2.3 (0.9–5.6)	2.2 (0.9–4.3)
Current cigarette smokers	56	-0.482 (0.177)**	13 (23.1)	2.0 (1.0–4.3)	1.9 (0.9–4.3)
Secondhand smoke/aerosol	45	0.011 (0.177)	7 (14.0)	1.4 (0.6–3.3)	1.2 (0.5–3.0)
Use other tobacco products	11	-0.524 (0.309)	1 (9.1)	0.8 (0.1–5.6)	0.9 (0.1–6.4)
Unexposed	97	0 (Referent)	11 (11.3)	Referent	1 (Referent)

* Model controlled for age and race/ethnicity.

** p<0.05.

As shown in the last two columns of Table 2, the risk of SGA in neonates of women who were current ENDS dual-users while pregnant was 23.5% (4/17), 33.3% (2/6) for ENDS-only users, and 23.1% (13/56) for non-ENDS users who were smokers; this is in contrast to 11.7% (12/103) for non-smokers/non-ENDS users who were not exposed to secondhand smoke or ENDS aerosols. The crude RR for SGA was 2.1 for ENDS dual users, 2.9 for ENDS only users, and 2.3 for dual and ENDS-only users combined. After adjusting for maternal age and race/ethnicity, the corresponding RR of SGA was 1.9 (95% CI: 0.6–5.5) for ENDS dual users, 3.1 (95% CI: 0.8–11.7) for ENDS-only users, and 1.9 (95% CI: 0.9–4.3) for cigarette smokers. Combining the two types of ENDS users (dual and ENDS-only), their risk of having an SGA neonate was 2.2 (95% CI: 0.9–4.3). Those reporting secondhand exposure to cigarette smoke or ENDS aerosols had a slightly increased risk of SGA (RR=1.2, 95% CI: 0.5–3.0). All 95% CIs included the null value.

Table 3 shows the same birth-weight outcomes as Table 2; however, it excludes 49 participants who self-reported not using any tobacco product and not having exposure to secondhand smoke or ENDS aerosols, but nevertheless had salivary cotinine or CO levels compatible with tobacco use or combustible tobacco. The differences in gender-specific and gestational-age-specific birth weight for ENDS users and smokers with respect to the referent category were slightly larger when those 49 exposed participants were removed from the referent (e.g. for current ENDS-only users, the difference of -0.498 became -0.540 units of standard deviation from the US referent population). Still, the only significant difference in birth weight z-score was for active cigarette smokers. However, the risk of SGA among ENDS-only users increased from 3.1 to 5.1 (95% CI: 1.2–22.2), and the combination of the two types of ENDS users (dual and ENDS-only), increased from 2.2 to almost a four-fold increased risk of SGA (RR=3.8, 95% CI: 1.3–11.2). Overall, the results of the exclusion of active tobacco users, from the referent group, resulted in larger estimates of the association because it was corrected for misclassification of the exposure, due to the error introduced by self-report (i.e. nondisclosure of smoking).

**Table 3:** Pregnancy outcomes of pregnant women according to ENDS and/or smoking status, excluding smokers who did not disclose their smoking from the referent, Little Rock, Arkansas, 2015–2017 (N=199)

*Self-reported tobacco use at baseline*	*n*	Multivariate* mean z-score birth weight difference (SE)	*Smallness-forgestational-age (%)*	*Smallness-forgestational-age crude risk ratio ( 95% CI)*	Smallness-forgestational-age multivariate* risk ratio ( 95% CI)
Current ENDS dual users	17	-0.303 (0.274)	4 (23.5)	2.8 (0.8–10.1)	2.5 (0.7–8.8)
Current ENDS-only users	6	-0.540 (0.417)	2 (33.3)	4.0 (0.9–17.4)	5.1 (1.2–22.2)
Any current ENDS use	23	-0.368 (0.243)	6 (26.1)	3.1 (1.0–10.0)	3.8 (1.3–11.2)
Current cigarette smokers	56	-0.490 (0.190)**	13 (23.1)	2.8 (1.0–8.0)	2.6 (0.9–7.2)
Secondhand smoke/aerosol	45	0.006 (0.190)	7 (14.0)	1.9 (0.6–5.9)	1.6 (0.6–4.8)
Use other tobacco products	11	-0.548 (0.317)	1 (9.1)	1.1 (0.1–8.8)	1.3 (0.2–10.7)
Unexposed	64	0 (Referent)	5 (7.8)	Referent	1 (Referent)

* Model included maternal age and race/ethnicity as covariates.

** p<0.05.

### Sensitivity analysis of risk of small-for-gestational-age by validity of self-reported tobacco use

Table 4 presents the range of values of the RR for SGA compatible with our data that corrected for the misclassification due to self-reports, using hair nicotine (≥2.77 ng/mL) as the standard. The input values for the table are the estimates of sensitivity and specificity that come from: a) our sub-study population in the companion publication^36^, and b) those estimates of sensitivity and specificity in studies published elsewhere^27^. The RR estimates for the association between ENDS use and the risk of SGA, based on expected values if there was no misclassification, were 3 to 4 times the uncorrected estimates. The corrected estimates of the RR were 6.5 and 8.5, using the observations on validity of self-report from our small study, or the larger validation study^27^, respectively.

**Table 4:** Sensitivity analysis of results of risk of having a small-for-gestational-age neonate, corrected for misclassification of exposure, Little Rock, Arkansas, 2015–2017 (N=120 from Table 2, N=23 exposed to ENDS, and N=97 self-reported as unexposed)

*Biomarker criteria versus self-report (type of users)*	Source of estimates of sensitivity and specificity (n*)	*Sensitivity of exposure*	*Specificity of exposure*	Any ENDS current use risk ratio** ( 95% CI)
Self-report	This study	Naïve	Naïve	2.3 (0.9–5.6)
Hair nicotine ≥2.77 ng/mL (smokers/ENDS vs unexposed)	This study (76)	0.83	0.85	6.5 (2.5–15.0)
Hair nicotine ≥2.77 ng/mL (smokers vs passive non-smokers)	Kim (289)	0.84	0.82	8.5 (3.3–19.5)

* Excludes secondhand exposure to smoke or aerosols from others for the reference category to estimate sensitivity and specificity.

** The sensitivity and specificity of the outcome (SGA) measurement is assumed to be perfect (i.e. 1.0).

## DISCUSSION

In this study population of a low-risk pregnancy clinic, consisting of low-income pregnant women residing in an ethnically diverse population, we estimated a 6.8% prevalence for current ENDS use during pregnancy. This is consistent with the 6.1% estimate of current ENDS-using adults from the 2014 Arkansas BRFSS^33^ and a national study of pregnant women^1^. Consistent with the latter source, we also found that 75.0% of ENDS users were current cigarette smokers.

We have completed the first assessment to date of the effects of ENDS use on reproductive outcomes, and although most of the pregnant women using these emerging tobacco products were also current smokers, the few observations among ENDS-only users indicate that their risk of SGA are not less than those of smokers. We also found that once the misclassification of self-report smoking status was removed, ENDS-only users had a significantly increased risk for having SGA neonates compared with non-smokers/non-ENDS users who were not exposed to secondhand smoke, ENDS aerosols, or other tobacco products. Because 50.5% (49/97) of the participants in a comparison group formed on the basis of self-reports (i.e. unexposed to tobacco, including secondhand smoke, ENDS aerosols, or other tobacco products) had levels of tobacco biomarkers that were consistent with active tobacco use, estimates based on questionnaire-only data were biased towards the null value, as shown by the difference between the naïve results in Table 2 and the corrected estimates of the association shown in Table 3. The corrected estimates are more likely to reflect actual exposure to tobacco, and more closely mirror the results of the sensitivity analysis in Table 4, which also addresses the misclassification by self-report, but using a measure of long-term exposure (hair nicotine).

Our results stress the importance of using valid biomarkers to measure tobacco use when assessing the impact of these products on reproductive outcomes, something discussed at length in the chapter on Reproductive Outcomes in the 2014 US Surgeon General’s Report^12^.

Since we found a change of the estimate of the association of ENDS with SGA by maternal age and race/ethnicity, we included the adjustment for these two factors and controlled for confounding by these factors. The analysis of SGA and z-score of birth weights are gestational-age specific, and hence the results are unlikely affected by gestational age. The size of the reported associations (i.e. SGA RR=5.1 and 3.8, for ENDS-only users and any current ENDS user, respectively) had large E-values, 9.7 and 7.1, indicating that the associations were robust to potential unmeasured or uncontrolled confounding^43^.

Both ENDS and cigarettes contain many fetotoxins, including CO, the most potent fetotoxin found in cigarette smoke^44^ but absent in ENDS aerosols. Because most ENDS users continued to smoke cigarettes, our data suggest that adding ENDS use to smoking does not reduce the risk for SGA. We found an increased risk of SGA for those who reported using ENDS.

### Limitations

The small number of observations is a limitation of this study. This report was based on the follow-up of 232 women, with only 77 having complete data on hair nicotine levels. A well-powered study to detect a 2-fold to 3-fold increase in risk of SGA, assuming a 12% risk of SGA among pregnant women not using ENDS or smoking (i.e. the referent group), would require about 300 participants per group (e.g. ENDS dual users, ENDS-only users, cigarette smokers, and the referent group).

We did consider maternal age, race/ethnicity and education as well as parity as potential confounders, and reported estimates that controlled for actual confounders, maternal age and race/ethnicity, but not other characteristics such as paternal age, parental weight and height, maternal prepregnancy body mass index, or other comorbidities to assess the effects of smoking and ENDS use, which could improve the assessment of reproductive risk^45^. We lack data to accurately measure differences that may exist regarding the dynamics of ENDS use and/or smoking during pregnancy. We found an association between earlier gestation and ENDS use, which could reflect the known dynamics of smoking cessation throughout pregnancy^46^.

We addressed the limitation of measuring smoking status and ENDS use by self-report only, which could have led to a bias towards the null value but was corrected for misclassification using biomarker data from our own study, i.e. salivary cotinine and exhaled CO. It is possible that repeated measurements of hair nicotine, salivary cotinine, and CO could increase the accuracy of classifying ENDS and smoking in future studies. Because our study population was selected using non-random sampling and was limited in size, our findings may not be generalizable.

## CONCLUSIONS

Our data show that 1 out of 15 pregnant women in the study population were using ENDS in the past month in 2015–2017, with 75% being concurrent cigarette smokers. We found that ENDS use during pregnancy increased the risk of SGA. We also showed that after correction for misclassification by self-report, the strength of the association of ENDS use and smoking and the risk of SGA is larger (RR = 6–8) than previously reported (RR = 2–3). Larger and well-funded epidemiologic studies on this topic are needed.
