# Supplementary material for: Use of Electronic Nicotine Delivery Systems (ENDS) by pregnant women I: Risk of small-for-gestational-age birth
Source: Tob Induc Dis. 2019 May 21;17:44. doi: 10.18332/tid/106089 (PMC6662791; doi:10.18332/tid/106089)
Supplement: Supplementary file 1 [file TID-17-44-s1.pdf]

Supplementary Table 1. Other pregnancy outcomes not considered in the analysis, among the 232 singleton live births of the 248 women enrolled in the study of ENDS and pregnancy outcomes, Little Rock, Arkansas, 2016–2017

Outcome	Number	Per cent
**Preterm delivery**	22	9.5
**Admissions to the neonatal intensive care unit**	2	0.8
**Congenital malformations**	3	1.3

© 2019 Cardenas V.M.
